# Zinc phosphate-based nanoparticles as a novel antibacterial agent: *in vivo* study on rats after dietary exposure

**DOI:** 10.1186/s40104-019-0319-8

**Published:** 2019-02-12

**Authors:** Pavel Horky, Sylvie Skalickova, Lenka Urbankova, Daria Baholet, Silvia Kociova, Zuzana Bytesnikova, Eliska Kabourkova, Zuzana Lackova, Natalia Cernei, Milica Gagic, Vedran Milosavljevic, Vendula Smolikova, Eva Vaclavkova, Pavel Nevrkla, Pavel Knot, Olga Krystofova, David Hynek, Pavel Kopel, Jiri Skladanka, Vojtech Adam, Kristyna Smerkova

**Affiliations:** 1 Department of Animal Nutrition and Forage Production, Mendel University in Brno, Zemedelska 1, CZ-613 00 Brno, Czech Republic; 2 Department of Chemistry and Biochemistry, Mendel University in Brno, Zemedelska 1, CZ-613 00 Brno, Czech Republic; 3 Central European Institute of Technology, Brno University of Technology, Purkynova 123, CZ-612 00 Brno, Czech Republic; 4 Institute of Animal Science, Komenskeho 1239, CZ-517 41 Kostelec nad Orlici, Czech Republic; 5 Department of Animal Breeding, Mendel University in Brno, Zemedelska 1, CZ-613 00 Brno, Czech Republic

**Keywords:** Aerobic bacteria, Antibiotics, Coliforms, Nanomaterials, Oxidative stress

## Abstract

**Background:**

Development of new nanomaterials that inhibit or kill bacteria is an important and timely research topic. For example, financial losses due to infectious diseases, such as diarrhea, are a major concern in livestock productions around the world. Antimicrobial nanoparticles (NPs) represent a promising alternative to antibiotics and may lower antibiotic use and consequently spread of antibiotic resistance traits among bacteria, including pathogens.

**Results:**

Four formulations of zinc nanoparticles (ZnA, ZnB, ZnC, and ZnD) based on phosphates with spherical (ZnA, ZnB) or irregular (ZnC, ZnD) morphology were prepared. The highest *in vitro* inhibitory effect of our NPs was observed against *Staphylococcus aureus* (inhibitory concentration values, IC_50_, ranged from 0.5 to 1.6 mmol/L)*,* followed by *Escherichia coli* (IC_50_ 0.8–1.5 mmol/L). In contrast, methicillin resistant *S. aureus* (IC_50_ 1.2–4.7 mmol/L) was least affected and this was similar to inhibitory patterns of commercial ZnO-based NPs and ZnO. After the successful *in vitro* testing, the in vivo study with rats based on dietary supplementation with zinc NPs was conducted. Four groups of rats were treated by 2,000 mg Zn/kg diet of ZnA, ZnB, ZnC, and ZnD, for comparison two groups were supplemented by 2,000 mg Zn/kg diet of ZnO-N and ZnO, and one group (control) was fed only by basal diet. The significantly higher (*P* < 0.05) Zn level in liver and kidney of all treated groups was found, nevertheless Zn NPs did not greatly influence antioxidant status of rats. However, the total aerobic and coliform bacterial population in rat feces significantly decreased (*P* < 0.05) in all zinc groups after 30 d of the treatment. Furthermore, when compared to the ZnO group, ZnA and ZnC nanoparticles reduced coliforms significantly more (*P* < 0.05).

**Conclusions:**

Our results demonstrate that phosphate-based zinc nanoparticles have the potential to act as antibiotic agents.

**Electronic supplementary material:**

The online version of this article (10.1186/s40104-019-0319-8) contains supplementary material, which is available to authorized users.

## Background

Zinc-based nanomaterials have been applied in several fields including agriculture, chemistry, textile and food industry, electronics, and medicine [1–7]. Due to their antibacterial activity, the Zn nanoparticles (NPs), particularly ZnO-based, have been designed and tested for utilization in veterinary and human medicine. Their bactericidal ability has been demonstrated against diverse bacterial species, including pathogens *Listeria monocytogenes*, *Escherichia coli*, *Staphylococcus aureus*, *Pseudomonas aeruginosa, Campylobacter jejuni, Salmonella enterica* [8–11] and antibiotic-resistant strains including methicillin resistant *S. aureus* (MRSA) and extended spectrum beta-lactamases producing *E. coli* and *Klebsiella pneumoniae* [12, 13]. Considering their substantial bactericidal potential, Zn nanoparticles represent promising alternatives to antibiotics or an enhancement of antibiotics against drug resistant bacteria [14].

The exact mode of action of Zn NPs is not well understood; however, one of the most plausible mechanisms comprises generation of reactive oxygen species (ROS). The highly reactive hydroxyl radicals are able to enter bacterial cells and damage them, while superoxide anions or hydrogen peroxide are considered less toxic [15, 16]. Another mechanism may involve disruption of the bacterial cell wall after the contact with nanoparticles [17, 18]. Furthermore, metal NPs are also able to induce different biological responses in eukaryotes [19, 20]. Due to ROS production, the ZnO NPs cause oxidative stress which leads to inflammation and even death of mammalian cell lines [21].

In the present study, four phosphate-based NPs formulations were synthesized and characterized. After confirmation of their antibacterial activity *in vitro*, the study on rats was conducted. The *in vivo* test aimed to determine whether the selected phosphate-based NPs have comparable effect to ZnO and ZnO NPs on the gut microbiome and on overall health of rats as a model organism.

## Methods

### Chemicals

All chemicals were purchased from Sigma Aldrich (St. Louis, MO, USA) and Penta (Prague, Czech Republic) of p.a. purity, unless noted otherwise. The pH value was measured using inoLab® Level 3 (Wissenschaftlich-Technische Werkstatten GmbH; Weilheim, Germany). Deionised water underwent demineralization by reverse osmosis using the instruments Aqua Osmotic 02 (Aqua Osmotic, Tisnov, Czech Republic) and it was subsequently purified using Millipore RG (Millipore Corp., Waltham, MA, USA) – 18 MΏ MilliQ water.

ZnO and ZnO-based NPs (ZnO-N) were supplied by Sigma-Aldrich. According to manufacturer, the average particle size was ≤100 nm (see Additional file 1: Figure S1).

### Zinc NPs synthesis

#### ZnA

Zn(NO_3_)_2_·6H_2_O (4.46 g) was dissolved in water (50 mL) and solution was heated to 60 °C. (NH_4_)_2_HPO_4_ (1.32 g in 20 mL of water) was added while stirring and white precipitate was immediately formed. The suspension was stirred for 2 h, cooled and water was added to reach 100 mL.

#### ZnB

Zinc phosphate was prepared as described above, only sodium salt Na_2_HPO_4_·7H_2_O (2.68 g) was used instead of ammonium salt.

#### ZnC

Zn(NO_3_)_2_·6H_2_O (3 g) was dissolved in water (50 mL) and solution was heated to 60 °C. Na_4_P_2_O_7_ (1.33 g in 20 mL of water) was added with stirring and white precipitate was immediately formed. The suspension was then prepared as described above.

#### ZnD

Zn(NO_3_)_2_·6H_2_O (1.49 g) dissolved in water (50 mL) was heated to 60 °C. Na_5_P_3_O_10_ (0.74 g in 20 mL of water) was added while stirring and white precipitate was also immediately formed. The suspension was then prepared as described above. All prepared Zn NPs were subjected to sedimentation and washed with water to remove byproducts and free zinc ions. Finally, the rest of water was removed by lyophilization. For *in vitro* experiments, the samples were resuspended in water and for *in vivo* experiment, the lyophilized particles were added directly to the diet.

### Particle size and structural analysis

The mean particle diameter and size distribution were determined by dynamic light scattering on a Malvern Zetasizer (NANO-ZS, Malvern Instruments Ltd., Worcestershire, UK).

Transmission electron microscopy (TEM) images of dried samples on copper grids were taken with a Tecnai F20 microscope (FEI, Eindhoven, Netherlands) at appropriate magnifications.

X-ray powder diffraction analysis (XRD) of zinc phosphates was carried out on D8 Advance ECO (Bruker, AXS GmbH, Karlsruhe, Germany). Bragg–Brentano geometry, CuKα radiation (λ = 1.54178 Å), the range of 2θ = 4–60° and room temperature were used for analysis.

### *In vitro* antibacterial testing

The antibacterial effect of Zn NPs and ZnO was analyzed by: a) counting of bacterial colonies, b) live/dead assay, and c) bacterial growth curves. *Escherichia coli* NCTC 13216, *Staphylococcus aureus* NCTC 8511, and Methicillin-resistant *S. aureus* CCM 7110 (Czech Collection of Microorganisms, Brno, Czech Republic) were cultured in Muller-Hinton (MH) broth (Oxoid, Hampshire, UK) overnight at 37 °C and shaking at 150 r/min.

#### Plating technique

Bacterial suspensions at concentrations of ∼1.0 × 10^8^ CFU/mL (measured by optical density at 600 nm) were serially diluted in tenfold steps in MH broth. A total of 900 μL diluted bacterial cultures were mixed with 100 μL of Zn NPs or ZnO at the final concentration of 5 mmol/L. After 2 h incubation at 37 °C, 100 μL of each inoculum was spread on MH agar and incubated for 24 h at 37 °C. The colony forming units (CFUs) were counted and compared and expressed in % change to that of control samples.

#### Live/dead assay

The MRSA culture was incubated with Zn NPs or ZnO (as described above) and then centrifuged and washed with 0.85% NaCl. For the live/dead assay, fluorescent dyes, SYTO9 (Thermo Fisher Scientific, USA) and propidium iodide (PI; Sigma Aldrich, St. Louis, USA) were used and bacterial cells were observed on Olympus IX71 inverted fluorescence microscope (Olympus, Tokyo, Japan). The excitation wavelength was 460–495 nm for SYTO9 and 545–580 nm for PI.

#### Growth curve method

One hundred μL of each bacterial suspension (∼1.0 × 10^6^ CFU/mL) was placed into a 96-well microplate and mixed with Zn NPs or ZnO in ratio 1:1 (total volume 200 μL). The bacterial growth was detected by Multiskan EX (Thermo Fisher Scientific, Bremen, Germany) using Ascent Software. The optical density reads at 620 nm were monitored at time zero, and then at 30 min intervals for 24 h at 37 °C.

### Animal feeding experiment

The experiments were performed with the approval of the Ethics Commission at the Faculty of AgriSciences, Mendel University in Brno, Czech Republic in accordance with Act No. 246/1992 Coll. for the protection of animals against cruelty. Throughout the experiment, microclimatic conditions were maintained at 23 ± 1 °C, 60% humidity, and the light regime (12 h L, 12 h D) with a maximum illumination of 200 lx. Laboratory male rats of the outbred strain Wistar albino were used as model animals. Animals were divided into seven groups of ten rats each. Rat average initial weight was 144 ± 2 g. Four groups of rats were fed with phosphate-based zinc nanoparticles (ZnA, ZnB, ZnC, ZnD) in the dose of 2,000 mg Zn/kg diet. Fifth group was fed by commercial zinc nanoparticles (ZnO-N) in the dose 2,000 mg Zn/kg diet. Sixth group was fed by ZnO in the dose of 2,000 mg Zn/kg diet. The last (control) group had no addition of Zn in their feed (C). Animals were weighed at regular intervals (day 0, 7, 14, 21 and 28). All groups of rats had mono diet (wheat) with 2.7 mg/kg of Zn. The experiment lasted for 28 d. The animals had access to feed and drinking water *ad libitum*. At the end of the experiment, five animals from each group were put to death and blood, kidney, duodenum and liver samples were dissected out and used for chemical analyses. Samples for histopathology were fixed using 10% formaldehyde.

### Zn determination by atomic absorption spectrometry

The digestion mixture was prepared in digestion vials and composed of 10.0 μL of blood mixed with nitric acid suprapure (300 μL) and 30% hydrogen peroxide (200 μL). Samples were digested in the Microwave 3000 (Anton Paar GmbH, Austria, with the power of 100 W) for 30 min at 140 °C. Homogenized liver and kidney (500 ± 0.1 mg) were decomposed using nitric acid suprapure (5 mL), 30% hydrogen peroxide (2 mL), and deionized water (3 mL) as digestion mixture in MW Ethos ONE (Milestone, Italy) for 30 min at 210 °C. Zn was determined by the 240FS AA (Agilent Technologies, USA) atomic absorption spectrometer with deuterium background correction. The instrument operated under conditions recommended by manufacturer with air-acethylene flame (flow rate 13.5 L/min and 2.0 L/min) and 213.86 nm resonance line.

### Oxidative status determination

#### Sample preparation

Two grams of liver or kidney from each animal were homogenized in a friction bow with the addition of liquid nitrogen and 1.5 mL of water. For blood, 200.0 μL of plasma with 0.5 mL of MilliQ was kept in liquid nitrogen for 2 min. After homogenization, each sample was sonicated using an ultrasound needle for 2 min, shaken for 10 min (blood for 1 min), and centrifuged for 20 min at 25,000×*g* at 4 °C. One hundred μL of supernatant from each sample were mixed with 100 μL of 10% TFA and centrifuged for 20 min at 25,000×*g* at 4 °C. Supernatant was collected for the antioxidant activity analysis.

The BS-400 automated spectrophotometer (Mindray, China) was used for oxidative status analysis, specifically for TEAC (Trolox equivalent antioxidant capacity), DPPH (2,2-diphenyl-1-picrylhydrazyl), MDA (Malondialdehyde), and SOD (Superoxide dismutase).

#### TEAC

ABTS (2,2′-azino-bis(3-ethylbenzothiazoline-6-sulphonic acid) (54.9 mg) was dissolved in 20.0 mL of phosphate buffer (pH 7.0; 5 mmol/L) and activated to cation of ABTS+ radical by addition of MnO_2_ (1.0 g) under occasional stirring for 30 min. Subsequently, 15.0 μL of sample were added. Absorbance of solution was measured at λ = 734 nm.

#### DPPH

A total of 150 μL of 0.095 mmol/L 2,2-diphenyl-1-picrylhydrazyl was transferred into plastic cuvette with 15.0 μL of sample. Absorbance was measured for 12 min at λ = 505 nm. To assess the production of free radicals, the difference in absorbance between the reagent with and without a sample was taken after the 10 min incubation period.

#### MDA

Trichloroacetic acid was used because of its ability to precipitate proteins, bilirubin, unsaturated fatty acids and lipoproteins. Each sample (300 μL) was mixed with 10 μL of 0.5 mol/L solution of butylated hydroxytoluene in 96% ethanol (*v/v*) and 310 μL of 20% trichloroacetic acid (*v/v*) prepared in 0.6 mol/L HCl. After 20 min incubation on ice, the mixture was centrifuged at 11,000×*g* for 15 min. Subsequently, 400 μL of the supernatant was mixed with 800 μL of 30 mmol/L thiobarbituric acid and the mixture was incubated at 90 °C for 30 min. After cooling on ice, MDA absorbance was measured at 535 nm and the concentration was subtracted from the calibration curve.

#### SOD

The SOD Assay Kit was used for the superoxide dismutase analysis. A total of 200 μL of the reagent R1 (WTS solution diluted 20 times in a buffer) was pipetted into a plastic cuvette and incubated at 37 °C for 1.8 min. Afterwards, each sample (20 μL) was added and incubated for 6.3 min. The reaction started by adding 20 μL of the reagent R2 (enzyme solution 167 times diluted in a buffer) and this was incubated for 72 s and absorbance was then measured at 450 nm.

#### Reduced glutathione analysis

The high-performance liquid chromatography with electrochemical detection (ESA Inc., Chelmsford, MA) was used for reduced glutathione (GSH) determination. Samples were analyzed in the chromatographic column with reverse phase Zorbax eclipse AAA C18 (Agilent Technologies, USA). The flow rate of mobile phase was 1.1 mL/min and mobile phase consisted of A: trifluoroacetic acid (80 mmol/L) and B: 100% methanol. Compounds (GSH) were eluted by following gradients: 0 → 1 min (4% B), 2 → 5 min (7% B), 6 → 10 min (98% B), 11 → 20 min (4% B). Detection was carried out at applied potential 900 mV.

### Histopathology analysis

Tissues were fixed individually in the 10% neutral buffered formaldehyde. Tissues sections were cut at 3.0 μm and placed onto Superfrost Plus slides (Leica, UK) with the orientation core placed up on the slide. All sections were oriented the same way and the entire tissue block was cut with remaining sections dipped in wax and stored at the room temperature. The sections were stained with hematoxylin and eosin following standard procedures. Photographs were taken using an inverted Olympus microscope IX 71 S8F-3 (Tokyo, Japan).

### Analysis of the total aerobic bacteria and coliforms in feces

The fecal samples were homogenized in sterile phosphate buffer solution (PBS) on ice (1:9 *w*/*v*) and the homogenate was serially diluted in PBS. Subsequently, 1.0 mL of diluted suspension was mixed with sterile molten Plate Count Agar (PCA) and MacConkey Agar (Sigma-Aldrich) in duplicates. The total colony counts from PCA and counts of coliforms from MacConkey Agar were enumerated after 24 h at 37 °C. The results are expressed as log (CFU/g) of feces.

### Descriptive statistics

The data were processed statistically using STATISTICA.CZ, version 12.0 (Czech Republic). The results were expressed as the mean ± standard deviation (SD). Statistical significance was determined using ANOVA and Scheffé’s test (one-way analysis). The analysis of total counts and coliforms in feces was performed using one-way ANOVA with *post-hoc* Dunnett’s C test specialized for unequal variances and unequal sample sizes (IBM SPSS Statistics 21, Version 21.0. Armonk, NY, USA). The differences with *P* < 0.05 were considered significant.

## Results

### Zn NPs characterization

**Fig. 1:**
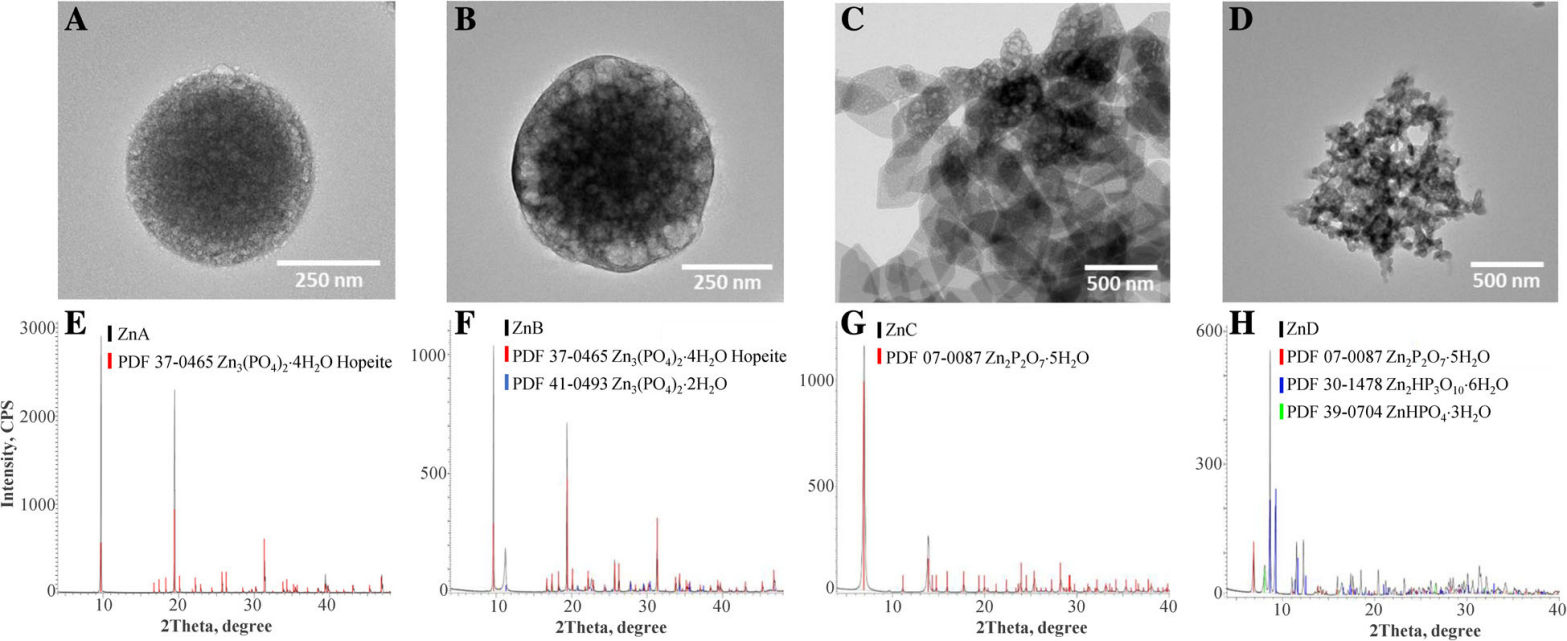
The phosphate-based zinc NPs size and structural characteristics. TEM images of **a** ZnA, **b** ZnB, **c** ZnC, and **d** ZnD. The XRD spectra of **e** ZnA, **f** ZnB, **g** ZnC, and **h** ZnD

In-house prepared particles were synthesized from various precursors – hydrogen phosphate (ZnA and ZnB), diphosphate (ZnC), and triphosphate (ZnD). ZnA and ZnB particles had spherical shape with the average diameter 477 and 521 nm, respectively (Fig. 1a and b). The other two types of particles, ZnC and ZnD, were based on diphosphate and triphosphate salts. The effect of anions was much greater than that of cations. ZnC and ZnD had irregular shape with tendency to form small aggregates (Fig. 1c and d). The determined average diameter was estimated to 452 (ZnC) and 1035 (ZnD) nm. The polydispersity indices of ZnA, ZnB and ZnC particles were between 0.16–0.19. The ZnD exhibited a higher polydispersity index (0.4) likely due to the formation of particles from the smaller parts (Fig. 1d).

Crystalline forms of samples allowed for their analysis by powder XRD and the identification of products by comparison with Powder Diffraction Files (PDF). For preparation of zinc phosphate, ammonium salts (ZnA) and sodium salts (ZnB), were used. From XRD spectrum of ZnA it follows that using ammonium salt only hopeite Zn_3_(PO_4_)_2_·4H_2_O (PDF 37–0465) was obtained (Fig. 1e). When sodium salt (ZnB) was used under the same conditions, 90% of product is hopeite but 10% was zinc phosphate dihydrate (PDF 41–0493, Fig. 1f). For ZnC preparation, diphosphate was applied and the product was identified as Zn_2_P_2_O_7_·5H_2_O (PDF 07–0087, Fig. 1g). More complicated situation occurred for ZnD. XRD analysis of the spectrum showed a mixture of three products (Fig. 1h). The most abundant component (60%) was as expected zinc triphosphate Zn_2_HP_3_O_10_·6H_2_O (PDF 30–1478); however, there was also Zn_2_P_2_O_7_·5H_2_O (07–0087) (30%) and ZnHPO_4_·3H_2_O (PDF 39–0704) (10%). Nevertheless, considering the method of sodium triphosphate preparation, the final product had satisfactory composition for biological testing.

### *In vitro* antibacterial activity

**Fig. 2:**
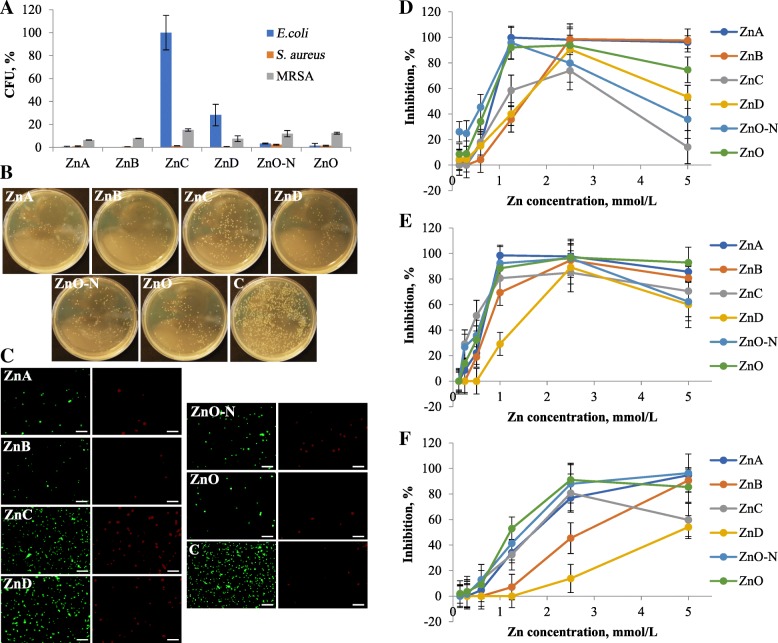
*In vitro* Zn NPs and ZnO antibacterial testing. **a** Bacterial counts after 24 h cultivation with 5 mmol/L zinc compounds. **b** Digital photographs of MRSA colonies on plates. **c** Fluorescence microscopy of live and dead cells (SYTO 9, green) and dead cells (PI, red), scale bar is 20 μm. The Zn NPs and ZnO inhibition effect on **d** *E. coli*, **e** *S. aureus*, and **f** MRSA (mean ± SD, *n* = 3)

The following analyses were performed with in-house prepared Zn NPs and commercial ZnO-N and ZnO as control standards. The *in vitro* antibacterial effect of NPs was verified by three different techniques and the results are shown in Fig. 2. First, counts of viable bacteria after the 24 h incubation period with 5 mmol/L Zn NPs and ZnO were determined (Fig. 2a). ZnA and ZnO greatly effected the growth of *E. coli* (99.6% inhibition with ZnA; 98.5% inhibition with ZnO) and ZnB inhibited *E. coli* completely. In contrast, ZnC had no effect on *E. coli* growth. All types of Zn NPs and ZnO caused > 97% inhibition of *S. aureus*. The spread-plate technique also showed that the reduction of MRSA was lower than that of *S. aureus* (Fig. 2b). The formation of viable colonies was suppressed after Zn NPs and ZnO exposure. The MRSA fluorescence images (Fig. 2c) are in a good agreement with the previous experiment. Images also demonstrate that ZnA, ZnB, and ZnO-N, and ZnO inhibited the growth of bacteria (green fluorescence). Although ZnC and ZnD did not significantly suppress bacterial proliferation, the amount of dead cells (red fluorescence) increased substantially.

The inhibitory effect (in %) of final Zn concentrations 0.15–5 mmol/L after 16 h was obtained (Fig. 2d–f) from the growth curves. The maximum inhibitory concentrations for *E. coli* (Fig. 2d) varied between 1.25–2.5 mmol/L. Interestingly, with the exception of ZnA and ZnB, the inhibitory effect of NPs decreased by increasing the Zn concentrations. In fact, the 5.0 mmol/L ZnC treatment had almost no effect on *E. coli* growth. For *S. aureus* (Fig. 2e), the highest inhibition was also observed in the range of 1.25–2.5 mmol/L of Zn, but the inhibition decline at 5 mmol/L was not as high as that for *E. coli*. Inhibition of MRSA (Fig. 2f) by Zn NPs (with the exception of ZnC and ZnO) were different from that of other bacterial strains. The overall comparison of antimicrobial activity using the 50% inhibitory concentration (IC_50_) is shown in Table 1.

**Table 1:** The IC_50_ values, mmol/L, comparison

	*E. coli*	*S. aureus*	MRSA
ZnA	0.90	0.68	1.60
ZnB	1.50	0.80	2.70
ZnC	1.10	0.48	1.60
ZnD	1.50	1.60	4.70
ZnO-N	0.70	0.62	1.45
ZnO	0.80	0.66	1.20

### Antioxidant status of rats

**Fig. 3:**
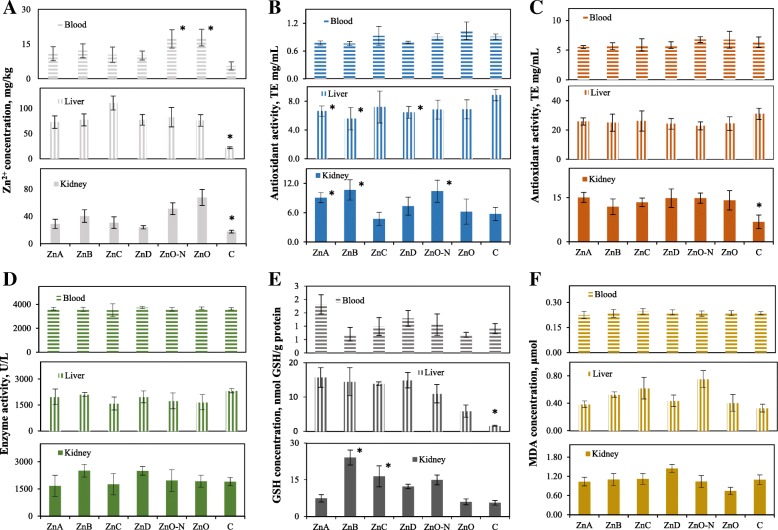
Levels of Zn and antioxidant system status in rat organism. **a** Zn concentration in blood, liver, and kidney. Antioxidant activities determined in blood, liver, and kidney using **b** DPPH and **c** TEAC methods. Results are expressed as mg/mL of Trolox equivalent. **d** Enzymatic activity of SOD. **e** Levels of GSH. Results are related to the protein content. **f** Concentration of MDA in blood, liver, kidney. *Mean values were significantly different (*P* < 0.05)

The levels of Zn, MDA, GSH, SOD, and overall antioxidant activity indicated the impact of Zn NPs and zinc oxide treatments on the rat liver, kidney, and blood (Fig. 3). The average Zn levels upon treatment were 75.2 ± 4.1 mg/kg in liver, 48.4 ± 6.2 mg/kg in kidney and 10.3 ± 4.1 mg/kg in blood (Fig. 3a). The liver and kidney Zn concentration significantly increased (*P* < 0.05) in all treated groups compared to non-treated control group. In blood, the significant increase (*P* < 0.05) of zinc level was observed only after treatment with ZnO-N and ZnO. Results of DPPH and TEAC assays were compared to antioxidant activity of the trolox equivalent as a standard. DPPH method showed increased antioxidant activity in kidney compared to that in blood and liver. Treatments with ZnA, ZnB, ZnO-N resulted in the significantly higher antioxidant activity in kidney when compared to that of the control (*P* < 0.05). In liver, the significant reduction (*P* < 0.05) of antioxidant activity was detected after treatments with ZnA, ZnB, ZnD in comparison to that of control (no treatment). The lowest antioxidant activity was revealed in the blood and this did not differ across samples of all Zn formulations (Fig. 3b). Based on TEAC method, the antioxidant activity of blood (5.8 ± 0.2 TE ng/mL) and liver (24.9 ± 3.3 TE ng/mL) was stable across all Zn formulations as well as controls. In kidneys (Fig. 3c), the antioxidant activity of all Zn variants was significantly (*P* < 0.05) higher (up to 14.4 ± 1.0 TE ng/mL) than in control (6.3 ± 1.1 TE ng/mL). The highest SOD activity was recorded in the blood at 4,000 ± 100 U/L. For liver and kidney, the enzyme activity was 2000 ± 500 U/L. Individual formulations of Zn did not influence the enzymatic activity of SOD when compared to that of samples with no Zn treatment (Fig. 3d). Levels of GSH reflected the whole protein concentration. In liver, GSH concentrations significantly (*P* < 0.05) increased in all Zn treated groups. Overall, it was clear that all Zn formulations had different effects on the GSH levels in the blood and kidney (Fig. 3e). MDA concentrations were 0.23 ± 0.05 μmol/L (blood), 1.2 ± 0.1 μmol/L (kidney), and 0.6 ± 0.1 μmol/L (liver) (Fig. 3f). No significant difference (*P* > 0.05) was detected.

### Histology of liver and duodenum and rat growth

The histological evaluation of the rat liver and small intestine (duodenum) was performed to assess possible negative effects of zinc treatments on these organs (see Additional file 1: Figure S2). Liver parenchyma of the rats fed with ZnA showed intact structure without necrosis and inflammation (Additional file 1: Figure S2A). Occasionally, the centrosocial districts of unstable steatal dystrophy were observed. Examination of the intestine showed the autolysis disintegration of the apical segments of the villus. Enterocytes were normal and more frequent occurrence of the cup cells was observed (Additional file 1: Figure S2B). In the ZnB group, heavy hepatodystrophy was seen in the liver. Portobilia was dilated with small groups of lymphocytic cellulization (Additional file 1: Figure S2C). In the intestine, the autologous disintegration of the apical segments of the villus (Additional file 1: Figure S2D) was also observed. Liver parenchyma of ZnC treated rats showed hepatodystrophy across the hepatic parenchyma (Additional file 1: Figure S2E). In intestine, deformed intestinal villi were observed, followed by an increase of epithelial cup cells and traces of inflammation (Additional file 1: Figure S2F) were visible in the stroma. Group of ZnD treated rats showed full-length hepatodystrophy in the liver parenchyma (Additional file 1: Figure S2G) and the intestine had hypertrophied and deformed villi. Many cup cells in the epithelium and numerous mitoses in the lining crypt were also seen (Additional file 1: Figure S2H). Treatment with commercial ZnO-N caused full-length, prolonged cholestatic hepatodystrophy in the liver (Additional file 1: Figure S2I). The intestine underwent focal autolytic disintegration of the mucosa and the villi were hypertrophied. In the lining of the crypts, a higher incidence of cup cells, numerous mitoses and mild inflammation in the cluster stroma were detected (Additional file 1: Figure S2J). Furthermore, the ZnO rat group exhibited a full-length mixed hepatodystrophy of the liver (Additional file 1: Figure S2K). The intestine in this group showed mild autolytic damage to the lining of the intestinal mucosa although intact regions were also found (Additional file 1: Figure S2L). Liver parenchyma of the control group (non-treated group) showed all-surface hepatodystrophy with significant portobiliary dilation (Additional file 1: Figure S2M). Intestinal samples showed destruction of apical parts of the cartilage, mild autolytic lesion damage, numerous cup cells in crypts, sparse and lymphocytic cellulization in the stroma of the cartilage (Additional file 1: Figure S2N).

Experimental rats were regularly weighted (0, 7, 14, 21, 28 d). The initial weight of all groups was in the range 130.0–155.0 g. The weight results were comparable in all groups both at the beginning and at the end of the experiment (see Additional file 1: Table S1). Only, the group ZnB showed an accelerated weight gain in comparison to that of all other groups at the end of the experiment.

### Effects of Zn NPs on total aerobic bacteria and coliforms in feces

**Fig. 4:**
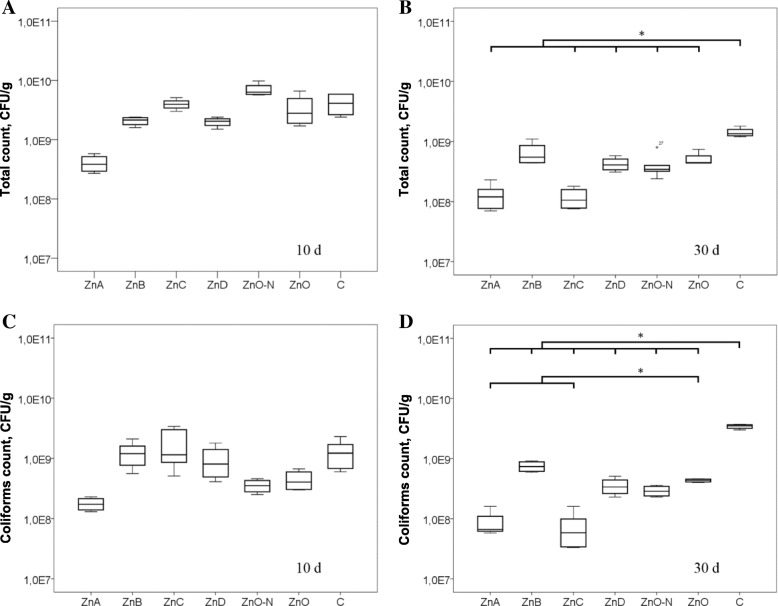
Effects of Zn NPs and ZnO on bacteria in feces. Total bacterial counts after **a** 10 d and **b** 30 d, counts of coliform bacteria after **c** 10 d and **d** 30 d of treatment (mean ± SD, *n* = 4). *Mean values were significantly different (*P* < 0.05)

The counts of total aerobic and coliform bacteria in rat feces at day 10 and day 30 of each treatment are shown in Fig. 4. After 10 d, the CFU levels from treated rats were not significantly different (*P* > 0.05) from that of the untreated control group. Interestingly, the ZnA group had the lowest CFU levels of both, total counts and coliforms. At day 30, all Zn NPs and ZnO treatments resulted in the significant decrease of the total aerobic and coliform bacteria (*P* < 0.05) compared to the control group, with the exception of ZnB in total aerobic bacteria (*P* > 0.05; Fig. 4b). Moreover, a significant decrease of coliforms (*P* < 0.05) was observed in rats exposed to ZnA and ZnC treatments compared to that of the ZnO group (Fig. 4d).

## Discussion

Zinc compounds are well known for their antimicrobial properties [15, 22, 23] where zinc-based antibacterial nanoparticles occupy a very important place. Recently, zinc oxide nanoparticles gained substantial scientific interest worldwide [24]. Their role as antibacterial agents has been studied in details in terms of particle size and morphology, surface modification, photocatalytic activity, and ROS generation [25–28]. However, there is some evidence that zinc phosphate-based materials show antibacterial effect as well [29, 30], and the combination with nanotechnologies could be useful. Here, we investigated antibacterial activity of zinc phosphate-based nanoparticles and compared them with commercial ZnO and ZnO NPs. We found that all four Zn NPs formulations inhibited bacterial growth *in vitro* of both, Gram-negative and Gram-positive bacteria. In some cases, the optical density measurements showed that high Zn concentrations (2.5–5 mmol/L) resulted in lower bacterial inhibition. This phenomenon was the most obvious for *E. coli* and this was also confirmed by the bacterial counts when the 5.0 mmol/L Zn concentration was used. The lower antibacterial response to high Zn concentrations may due to reduced solubility [31]. The low Zn doses can be even beneficial for bacterial growth and used as essential trace elements [32, 33]. Consequently, determination of the appropriate Zn NPs dosage is crucial for further applications. The different IC_50_ values between Gram-negative and Gram-positive bacteria implies interactions of zinc-based nanoparticles with the bacterial cell wall [34]. Moreover, the Gram-negative bacteria possess, in addition to a complex and poorly penetrable outer membrane, an advanced efflux system for removal of foreign compounds [35]. Interestingly, the lowest sensitivity to Zn NPs was found in MRSA. Unlike susceptible *S. aureus*, the MRSA genome contains the unique staphylococcal chromosomal cassette element SCC*mec*. This mobile genetic element includes *mec* genes, that encode resistance to β-lactams antibiotics [36, 37]. Importantly, the SCC*mec* cassette is also associated with metal resistance and there are several studies describing the presence of the zinc resistance gene (*czrC*) on the SCC*mec* element [38–40].

The antioxidant status of rat kidney, liver and blood was determined after zinc NPs treatments. In general, zinc acts as an antioxidant in an organism. The highest zinc accumulation was observed in liver, which plays a crucial role in zinc homeostasis regulation [41, 42]. The Zn concentrations in liver and kidney elevated after the treatment with all Zn NPs formulations. This is in line with the results presented by Baek et al., who found the highest accumulation of ZnO NPs in liver and kidney without any influence of rat gender or NPs size [43]. Moreover, this phenomenon (distribution of Zn NPs into liver and kidney after oral administration) has been described in a number of studies [44–46]. The total antioxidant activity was monitored by TEAC and DPPH methods. Our results confirmed that the most antioxidant activity was in kidney and liver, where the most important antioxidant processes take place. Jemai et al. [47] observed an increase of antioxidant activity in rat organs after zinc administration in concentration 4.0 mg/kg of body mass. Our study shows an increase of antioxidant activity compared to that of control by ZnA, ZnB, ZnO-N in liver and kidney. SOD activity was constant across different zinc formulations. These data correlate with the concentration of glutathione (GSH). It is assumed that normal range SOD and GSH is 7–22 U/mg and 20–30 nmol/L, respectively [48]. Another indicator of oxidative stress was the concentration of MDA where significantly elevated values were recorded after ZnC and ZnO-N treatments. All other values measured in blood, liver and kidneys were normal. Overall, it is clear that zinc formulations did not influence the rat antioxidant status. Our results are in concordance with Liu et al. [49]. On the contrary, Zn NPs are known to be capable of ROS production leading to oxidative stress [50–52]. A strong relationship between released Zn ions and ROS induced cytotoxicity has been reported in the literature [53–55]. Histological analysis showed the flat liver damage in treated and non-treated control rats. This phenomenon was likely caused by the mono diet or stress. The most damage was observed in a group of animals fed with ZnO-N and ZnC, although recent studies revealed that zinc and zinc nanoparticles have some protective effect on the liver [56–59].

To investigate Zn NPs effect on host-colonizing bacteria, the population of total aerobic bacteria and coliforms in rat feces was analyzed. Lee and co-workers tracked orally administered ZnO NPs and after few hours of the exposure, the particles were mainly localized in the rat gastrointestinal tract [60]. Therefore, Zn NPs should be able to influence gut microbiota and the interaction of nanoparticles with the gut microbial community is discussed in several recent studies [61–64]. Our study demonstrated that dietary supplementation of rats with phosphate-based Zn NPs altered the bacterial population in feces as well. Due to inconsistent results in the control group the bacterial count decline was not significant at day 10; however, over time, the bacterial count was clearly reduced. Besides the number of bacteria, administration to Zn NPs affected the intestinal microbiome diversity, as well [65]. This phenomenon is in agreement with the work by Feng et al., suggesting that the ileal bacterial community richness decreased in response to higher dose of ZnO NPs (100 mg/kg), and that *Lactobacillus* genus was reduced in particular [66]. On the contrary, Li et al. pointed out, that ZnO NPs could act anti-inflammatory in a dose-dependent manner. This may be associated with reduction of infection-causing bacteria and, vice versa, gain of probiotics (*Lactobacillus* and *Bifidobacterium*) in colon [67].

## Conclusions

In this study, four formulations of phosphate-based zinc nanoparticles were synthetized and tested. All four formulations exhibited antibacterial activity against Gram-positive and Gram-negative bacteria. The *in vivo* study on rats confirmed that in-house prepared zinc nanoparticles did not cause oxidative stress and changes in liver and duodenum tissues were comparable to that of the group treated with commonly used ZnO. The effect our Zn formulations on the rat microbiome was similar to that caused by ZnO. In fact, ZnA and ZnC nanoparticles caused even greater inhibition of coliform bacteria than ZnO. Therefore, these nanoparticles have a potential to be used as new antibacterial agents, especially for reduction of coliform bacteria. Further studies, primarily focused on Zn NPs applications in livestock productions, are warranted.

## Additional file

Additional file 1: **Figure S1.** TEM image of commercial ZnO-N. **Figure S2.** Histological analysis of liver (1) and duodenum (2) of the groups rats ZnA (A, B); ZnB (C, D); ZnC (E, F); ZnD (G, H); ZnO-N (I, J); ZnO (K, L) and control group (M, N). **Table S1.** The weight of rats, g. (DOCX 2889 kb)

## Abbreviations

ABTS: 2,2′-azino-bis(3-ethylbenzothiazoline-6-sulphonic acid)
C: Control
CFU: Colony forming unit
DPPH: 2,2-diphenyl-1-picrylhydrazyl
GSH: Reduced glutathione
MDA: Malondialdehyde
MH: Muller-Hinton
MRSA: Methicillin resistant *Staphylococcus aureus*
NPs: Nanoparticles
PBS: Phosphate buffer solution
PCA: Plate count agar
PDF: Powder diffraction files
PI: Propidium iodide
ROS: Reactive oxygen species
SCC: Staphylococcal cassette chromosome element
SOD: Superoxide dismutase
TEAC: Trolox equivalent antioxidant capacity
TEM: Transmission electron microscopy
XRD: X-ray powder diffraction

## References

[1] Dapkekar A, Deshpande P, Oak MD, Paknikar KM, Rajwade JM (2018). Zinc use efficiency is enhanced in wheat through nanofertilization. Sci Rep.

[2] Hagedorn K, Li WY, Liang QJ, Dilger S, Noebels M, Wagner MR (2016). Catalytically doped semiconductors for chemical gas sensing: aerogel-like aluminum-containing zinc oxide materials prepared in the gas phase. Adv Funct Mater.

[3] Shaheen TI, El-Naggar ME, Abdelgawad AM, Hebeish A (2016). Durable antibacterial and UV protections of in situ synthesized zinc oxide nanoparticles onto cotton fabrics. Int J Biol Macromol.

[4] Al-Naamani L, Dobretsov S, Dutta J (2016). Chitosan-zinc oxide nanoparticle composite coating for active food packaging applications. Innov Food Sci Emerg Technol.

[5] Javed MS, Chen J, Chen L, Xi Y, Zhang CL, Wan BY (2016). Flexible full-solid state supercapacitors based on zinc sulfide spheres growing on carbon textile with superior charge storage. J Mater Chem A.

[6] Wang C, Zhang LG, Su WP, Ying ZX, He JT, Zhang LL (2017). Zinc oxide nanoparticles as a substitute for zinc oxide or colistin sulfate: effects on growth, serum enzymes, zinc deposition, intestinal morphology and epithelial barrier in weaned piglets. PLoS One.

[7] Kaviyarasu K, Geetha N, Kanimozhi K, Magdalane CM, Sivaranjani S, Ayeshamariam A, et al. *In vitro* cytotoxicity effect and antibacterial performance of human lung epithelial cells A549 activity of zinc oxide doped TiO_2_ nanocrystals: investigation of bio-medical application by chemical method. Mater Sci Eng C-Mater Biol Appl. 2017;74:325–33. 10.1016/j.msec.2016.12.024 28254301

[8] Shankar S, Rhim JW (2017). Facile approach for large-scale production of metal and metal oxide nanoparticles and preparation of antibacterial cotton pads. Carbohydr Polym.

[9] Oun AA, Rhim JW (2017). Carrageenan-based hydrogels and films: effect of ZnO and CuO nanoparticles on the physical, mechanical, and antimicrobial properties. Food Hydrocoll.

[10] Premanathan M, Karthikeyan K, Jeyasubramanian K, Manivannan G. Selective toxicity of ZnO nanoparticles toward Gram-positive bacteria and cancer cells by apoptosis through lipid peroxidation. Nanomed-Nanotechnol Biol Med. 2011;7(2):184–92. 10.1016/j.nano.2010.10.001 21034861

[11] Xie YP, He YP, Irwin PL, Jin T, Shi XM. Antibacterial activity and mechanism of action of zinc oxide nanoparticles against *Campylobacter jejuni*. Appl Environ Microbiol. 2011;77(7):2325–31. 10.1128/AEM.02149-10 PMC3067441 21296935

[12] Alves MM, Bouchami O, Tavares A, Cordoba L, Santos CF, Miragaia M, et al. New insights into antibiofilm effect of a nanosized ZnO coating against the pathogenic methicillin resistant* Staphylococcus aureus*. ACS Appl Mater Interfaces. 2017;9(34):28157–67. 10.1021/acsami.7b02320 28782933

[13] Hameed ASH, Karthikeyan C, Ahamed AP, Thajuddin N, Alharbi NS, Alharbi SA, et al. *In vitr*o antibacterial activity of ZnO and Nd doped ZnO nanoparticles against ESBL producing *Escherichia coli* and *Klebsiella pneumoniae*. Sci Rep. 2016;6:11. 10.1038/srep24312 PMC4829841 27071382

[14] Chen CW, Hsu CY, Lai SM, Syu WJ, Wang TY, Lai PS (2014). Metal nanobullets for multidrug resistant bacteria and biofilms. Adv Drug Deliv Rev.

[15] Abu Ali H, Shalash AM, Akkawi M, Jaber S. Synthesis, characterization and *in vitro* biological activity of new zinc (II) complexes of the nonsteroidal anti-inflammatory drug sulindac and nitrogen-donor ligands. Appl Organomet Chem. 2017;31(11):14.

[16] Jiang YH, Zhang LL, Wen DS, Ding YL. Role of physical and chemical interactions in the antibacterial behavior of ZnO nanoparticles against* E. Coli*. Mater Sci Eng C-Mater Biol Appl. 2016;69:1361–6. 10.1016/j.msec.2016.08.044 27612837

[17] Cai Q, Gao YY, Gao TY, Lan S, Simalou O, Zhou XY, et al. Insight into biological effects of zinc oxide nanoflowers on bacteria: why morphology matters. ACS Appl Mater Interfaces. 2016;8(16):10109–20. 10.1021/acsami.5b11573 27042940

[18] Jain A, Bhargava R, Poddar P. Probing interaction of gram-positive and Gram-negative bacterial cells with ZnO nanorods. Mater Sci Eng C-Mater Biol Appl. 2013;33(3):1247–53. 10.1016/j.msec.2012.12.019 23827568

[19] Heim J, Felder E, Tahir MN, Kaltbeitzel A, Heinrich UR, Brochhausen C (2015). Genotoxic effects of zinc oxide nanoparticles. Nanoscale.

[20] Bondarenko O, Juganson K, Ivask A, Kasemets K, Mortimer M, Kahru A. Toxicity of ag, CuO and ZnO nanoparticles to selected environmentally relevant test organisms and mammalian cells *in vitro*: a critical review. Arch Toxicol. 2013;87(7):1181–200. 10.1007/s00204-013-1079-4 PMC3677982 23728526

[21] Xia T, Kovochich M, Liong M, Madler L, Gilbert B, Shi HB (2008). Comparison of the mechanism of toxicity of zinc oxide and cerium oxide nanoparticles based on dissolution and oxidative stress properties. ACS Nano.

[22] Jiao LF, Lin FH, Cao ST, Wang CC, Wu H, Shu MA (2017). Preparation, characterization, antimicrobial and cytotoxicity studies of copper/zinc-loaded montmorillonite. J Anim Sci Biotechnol.

[23] Pati R, Sahu R, Panda J, Sonawane A (2016). Encapsulation of zinc-rifampicin complex into transferrin-conjugated silver quantum-dots improves its antimycobacterial activity and stability and facilitates drug delivery into macrophages. Sci Rep.

[24] Sirelkhatim A, Mahmud S, Seeni A, Kaus NHM, Ann LC, Bakhori SKM (2015). Review on zinc oxide nanoparticles: antibacterial activity and toxicity mechanism. Nano-Micro Lett.

[25] Raghupathi KR, Koodali RT, Manna AC (2011). Size-dependent bacterial growth inhibition and mechanism of antibacterial activity of zinc oxide nanoparticles. Langmuir.

[26] Gupta A, Srivastava R (2018). Zinc oxide nanoleaves: a scalable disperser-assisted sonochemical approach for synthesis and an antibacterial application. Ultrason Sonochem.

[27] Yu HY, Chen GY, Wang YB, Yao JM (2015). A facile one-pot route for preparing cellulose nanocrystal/zinc oxide nanohybrids with high antibacterial and photocatalytic activity. Cellulose.

[28] Mishra PK, Mishra H, Ekielski A, Talegaonkar S, Vaidya B (2017). Zinc oxide nanoparticles: a promising nanomaterial for biomedical applications. Drug Discov Today.

[29] Dastjerdie EV, Oskoui M, Sayanjali E, Tabatabaei FS. *In-vitro* comparison of the antimicrobial properties of glass ionomer cements with zinc phosphate cements. Iran J Pharm Res. 2012;11(1):77–82. PMC3876566 25317187

[30] Chou AHK, LeGeros RZ, Chen Z, Li YH (2007). Antibacterial effect of zinc phosphate mineralized guided bone regeneration membranes. Implant Dent.

[31] Roguska A, Belcarz A, Pisarek M, Ginalska G, Lewandowska M. TiO_2_ nanotube composite layers as delivery system for ZnO and Ag nanoparticles - An unexpected overdose effect decreasing their antibacterial efficacy. Mater Sci Eng C-Mater Biol Appl. 2015;51:158–66. 10.1016/j.msec.2015.02.046 25842121

[32] Gielda LM, DiRita VJ. Zinc Competition among the Intestinal Microbiota. mBio. 2012;3(4):00171–12. 10.1128/mBio.00171-12 PMC3419517 22851657

[33] Lopez CA, Skaar EP (2018). The impact of dietary transition metals on host-bacterial interactions. Cell Host Microbe.

[34] Vijayalakshmi K, Sivaraj D (2015). Enhanced antibacterial activity of Cr doped ZnO nanorods synthesized using microwave processing. RSC Adv.

[35] Winterhalter M, Ceccarelli M. Physical methods to quantify small antibiotic molecules uptake into Gram-negative bacteria. Eur J Pharm Biopharm. 2015;95:63–7. 10.1016/j.ejpb.2015.05.006 26036449

[36] Shore AC, Coleman DC. Staphylococcal cassette chromosome *mec*: recent advances and new insights. Int J Med Microbiol. 2013;303(6–7):350–9. 10.1016/j.ijmm.2013.02.002 23499303

[37] Stefani S, Chung DR, Lindsay JA, Friedrich AW, Kearns AM, Westh H, et al. Meticillin-resistant *Staphylococcus aureus* (MRSA): global epidemiology and harmonisation of typing methods. Int J Antimicrob Agents. 2012;39(4):273–82. 10.1016/j.ijantimicag.2011.09.030 22230333

[38] Cavaco LM, Hasman H, Aarestrup FM. Zinc resistance of *Staphylococcus aureus* of animal origin is strongly associated with methicillin resistance. Vet Microbiol. 2011;150(3–4):344–8. 10.1016/j.vetmic.2011.02.014 21411247

[39] Hau SJ, Frana T, Sun J, Davies PR, Nicholson TL. Zinc resistance within swine-associated methicillin-resistant *Staphylococcus aureus* isolates in the United States is associated with multilocus sequence type lineage. Appl Environ Microbiol. 2017;83(15):9. 10.1128/AEM.00756-17 PMC5514667 28526788

[40] Argudin MA, Lauzat B, Kraushaar B, Alba P, Agerso Y, Cavaco L, et al. Heavy metal and disinfectant resistance genes among livestock-associated methicillin-resistant *Staphylococcus aureus* isolates. Vet Microbiol. 2016;191:88–95. 10.1016/j.vetmic.2016.06.004 27374912

[41] Olechnowicz J, Tinkov A, Skalny A, Suliburska J (2018). Zinc status is associated with inflammation, oxidative stress, lipid, and glucose metabolism. J Physiol Sci.

[42] Bondzio A, Pieper R, Gabler C, Weise C, Schulze P, Zentek J (2013). Feeding low or pharmacological concentrations of zinc oxide changes the hepatic proteome profiles in weaned piglets. PLoS One.

[43] Baek M, Chung HE, Yu J, Lee JA, Kim TH, Oh JM (2012). Pharmacokinetics, tissue distribution, and excretion of zinc oxide nanoparticles. Int J Nanomedicine.

[44] Paek HJ, Lee YJ, Chung HE, Yoo NH, Lee JA, Kim MK (2013). Modulation of the pharmacokinetics of zinc oxide nanoparticles and their fates in vivo. Nanoscale.

[45] Cho WS, Kang BC, Lee JK, Jeong J, Che JH, Seok SH (2013). Comparative absorption, distribution, and excretion of titanium dioxide and zinc oxide nanoparticles after repeated oral administration. Part Fibre Toxicol.

[46] Wang C, Lu JJ, Zhou L, Li J, Xu JM, Li WJ, et al. Effects of long-term exposure to zinc oxide nanoparticles on development, zinc metabolism and biodistribution of minerals (Zn, Fe, Cu, Mn) in mice. PLoS One. 2016;11(10):14. 10.1371/journal.pone.0164434 PMC5061426 27732669

[47] Jemai H, Messaoudi I, Chaouch A, Kerkeni A (2007). Protective effect of zinc supplementation on blood antioxidant defense system in rats exposed to cadmium. J Trace Elem in Med Bio.

[48] Gu YH, Zhao Z (2015). Significance of the changes occurring in the levels of interleukins, SOD and MDA in rat pulmonary tissue following exposure to different altitudes and exposure times. Exp Ther Med.

[49] Liu JH, Ma X, Xu YY, Tang H, Yang ST, Yang YF (2017). Low toxicity and accumulation of zinc oxide nanoparticles in mice after 270-day consecutive dietary supplementation. Toxicol Res.

[50] Pati R, Das I, Mehta RK, Sahu R, Sonawane A. Zinc-oxide nanoparticles exhibit genotoxic, clastogenic, cytotoxic and actin depolymerization effects by inducing oxidative stress responses in macrophages and adult mice. Toxicol Sci. 2016;150(2):454–72. 10.1093/toxsci/kfw010 26794139

[51] Syama S, Sreekanth PJ, Varma HK, Mohanan PV (2014). Zinc oxide nanoparticles induced oxidative stress in mouse bone marrow mesenchymal stem cells. Toxicol Mech Methods.

[52] Arakha M, Roy J, Nayak PS, Mallick B, Jha S (2017). Zinc oxide nanoparticle energy band gap reduction triggers the oxidative stress resulting into autophagy-mediated apoptotic cell death. Free Radic Biol Med.

[53] Hou J, Wu YZ, Li X, Wei BB, Li SG, Wang XK (2018). Toxic effects of different types of zinc oxide nanoparticles on algae, plants, invertebrates, vertebrates and microorganisms. Chemosphere.

[54] Shin YJ, Lee WM, Kwak JI, An YJ (2018). Dissolution of zinc oxide nanoparticles in exposure media of algae, daphnia, and fish embryos for nanotoxicological testing. Chem Ecol.

[55] Brun NR, Lenz M, Wehrli B, Fent K (2014). Comparative effects of zinc oxide nanoparticles and dissolved zinc on zebrafish embryos and eleuthero-embryos: importance of zinc ions. Sci Total Environ.

[56] Jihen E, Imed M, Fatima H, Abdelhamid K. Protective effects of selenium (Se) and zinc (Zn) on cadmium (Cd) toxicity in the liver and kidney of the rat: histology and cd accumulation. Food Chem Toxicol. 2008;46(11):3522–7. 10.1016/j.fct.2008.08.037 18824208

[57] Chatzicharalampous C, Jeelani R, Mikhael S, Aldhaheri S, Najeemudin S, Morris RT (2018). Zinc: An essential metal for maintenance of female fertility. Fertil Steril.

[58] Kumar N, Krishnani KK, Kumar P, Singh NP. Zinc nanoparticles potentiates thermal tolerance and cellular stress protection of *Pangasius hypophthalmus* reared under multiple stressors. J Therm Biol. 2017;70:61–8. 10.1016/j.jtherbio.2017.10.003 29108559

[59] Torabi F, Shafaroudi MM, Rezaei N (2017). Combined protective effect of zinc oxide nanoparticles and melatonin on cyclophosphamide-induced toxicity in testicular histology and sperm parameters in adult Wistar rats. Int J Reprod Biomed.

[60] Lee CM, Jeong HJ, Yun KN, Kim DW, Sohn MH, Lee JK (2012). Optical imaging to trace near infrared fluorescent zinc oxide nanoparticles following oral exposure. Int J Nanomedicine.

[61] Pietroiusti A, Magrini A, Campagnolo L (2016). New frontiers in nanotoxicology: gut microbiota/microbiome-mediated effects of engineered nanomaterials. Toxicol Appl Pharmacol.

[62] Westmeier D, Hahlbrock A, Reinhardt C, Frohlich-Nowoisky J, Wessler S, Vallet C (2018). Nanomaterial-microbe cross-talk: physicochemical principles and (patho) biological consequences. Chem Soc Rev.

[63] Mercier-Bonin M, Despax B, Raynaud P, Houdeau E, Thomas M (2018). Mucus and microbiota as emerging players in gut nanotoxicology: the example of dietary silver and titanium dioxide nanoparticles. Crit Rev Food Sci Nutr.

[64] Qiu KY, Durham PG, Anselmo AC (2018). Inorganic nanoparticles and the microbiome. Nano Res.

[65] Yausheva E, Miroshnikov S, Sizova E (2018). Intestinal microbiome of broiler chickens after use of nanoparticles and metal salts. Environ Sci Pollut Res.

[66] Feng YN, Min LJ, Zhang WD, Liu J, Hou ZM, Chu MQ, et al. Zinc oxide nanoparticles influence microflora in ileal digesta and correlate well with blood metabolites. Front Microbiol. 2017;8:10. 10.3389/fmicb.2017.00992 PMC5454036 28626453

[67] Li JQ, Chen HQ, Wang B, Cai CX, Yang X, Chai ZF (2017). ZnO nanoparticles act as supportive therapy in DSS-induced ulcerative colitis in mice by maintaining gut homeostasis and activating Nrf2 signaling. Sci Rep.

