# Supplementary material for: Zinc phosphate-based nanoparticles as a novel antibacterial agent: in vivo study on rats after dietary exposure
Source: J Anim Sci Biotechnol. 2019 Feb 12;10:17. doi: 10.1186/s40104-019-0319-8 (PMC6373129; doi:10.1186/s40104-019-0319-8)
Supplement: Supplementary file 1 — Figure S1. TEM image of commercial ZnO-N. Figure S2. Histological analysis of liver (1) and duodenum (2) of the groups rats ZnA (A, B); ZnB (C, D); ZnC (E, F); ZnD (G, H); ZnO-N (I, J); ZnO (K, L) and control group (M, N). Table S1. The weight of rats, g. (DOCX 2889 kb) [file 40104_2019_319_MOESM1_ESM.docx]

**Additional file**

**Zinc phosphate-based nanoparticles as a novel antibacterial agent: *In vivo* study on rats after dietary exposure**

Pavel Horky^a^, Sylvie Skalickova^a^, Lenka Urbankova^a^, Daria Baholet^a^, Silvia Kociova^b^, Zuzana Bytesnikova^b^, Eliska Kabourkova^b^, Zuzana Lackova^b,c^, Natalia Cernei^b,c^, Milica Gagic^b^, Vedran Milosavljevic^b,c^, Vendula Smolikova^b,c^, Eva Vaclavkova^d^, Pavel Nevrkla^e^, Pavel Knot^a^, Olga Krystofova^b,c^, David Hynek^b,c^, Pavel Kopel^b,c^, Jiri Skladanka^a^, Vojtech Adam^b,c^, Kristyna Smerkova^b,c^*^*^*

*^a^Department of Animal Nutrition and Forage Production, Mendel University in Brno, Zemedelska 1, CZ-613 00 Brno, Czech Republic; pavel.horky@mendelu.cz, sylvie.skalickova@gmail.com, lenka.urbankova@mendelu.cz, xbaholet@mendelu.cz,* *pavel.knot@mendelu.cz, jiri.skladanka@mendelu.cz*

*^b^Department of Chemistry and Biochemistry, Mendel University in Brno, Zemedelska 1, CZ-613 00 Brno, Czech Republic; skociova@gmail.com, zuzka.bytesnikova@gmail.com, eliska.kabourkova404@gmail.com, Lackova14@seznam.cz, cernei.natalia3@gmail.com, gagic.milica@gmail.com, grizlidripac@gmail.com, VendulaSmolikova@seznam.cz, olga.krystofova@seznam.cz, d.hynek@email.cz, paulko@centrum.cz,* *vojtech.adam@mendelu.cz, kristyna.smerkova@mendelu.cz*

*^c^Central European Institute of Technology, Brno University of Technology, Purkynova 123, CZ-612 00 Brno, Czech Republic*

*^d^Institute of Animal Science, Komenskeho 1239, CZ-517 41 Kostelec nad Orlici, Czech Republic; vaclavkova.eva@vuzv.cz*

*^e^Department of Animal Breeding, Mendel University in Brno, Zemedelska 1, CZ-613 00 Brno, Czech Republic; nevrkla.uchhz@mendelu.cz*

*Corresponding author: Kristyna Smerkova, Department of Chemistry and Biochemistry, Mendel University in Brno, Zemedelska 1, CZ-613 00 Brno, Czech Republic, E-mail: kristyna.smerkova@mendelu.cz; Phone: +420 545 133 290

**Figure S1** TEM image of commercial ZnO-N


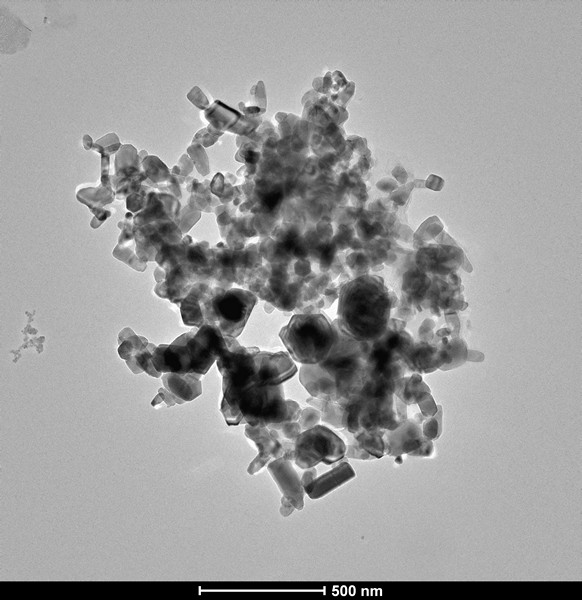


**Figure S2** Histological analysis of liver (1) and duodenum (2) of the groups rats ZnA (A, B); ZnB (C, D); ZnC (E, F); ZnD (G, H); ZnO-N (I, J); ZnO (K, L) and control group (M, N).


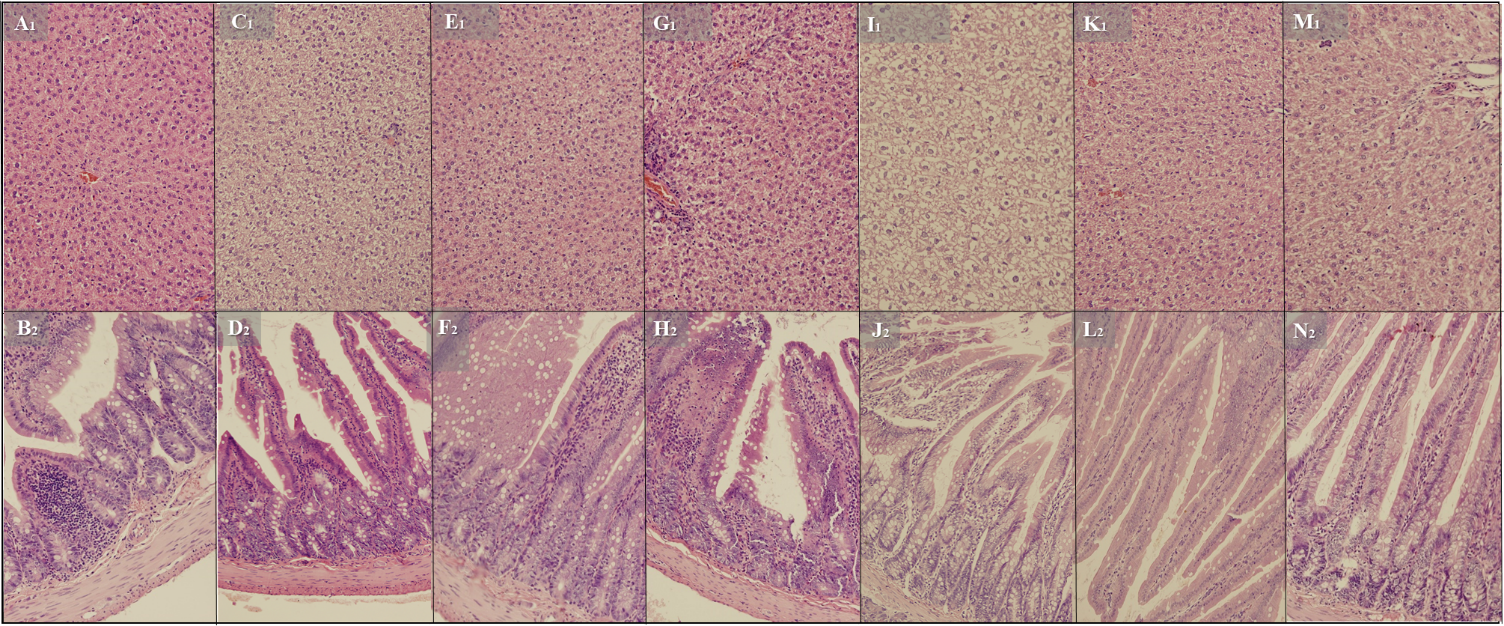


**Table S1** The weight of rats, g

| *Days of experiment* | | | | | |
| --- | --- | --- | --- | --- | --- |
|  | 0 | 7 | 14 | 21 | 28 |
| ZnA | 138.2±6.8 | 149.4±5.3 | 165.6±5.3 | 190.8±6.2 | 193.8±6.2 |
| ZnB | 137.0±4.5 | 162.8±7.2 | 194.4±8.6 | 208.0±6.6 | 218.2±6.4 |
| ZnC | 144.0±4.8 | 156.2±4.2 | 187.4±5.3 | 194.6±6.5 | 200.4±8.8 |
| ZnD | 130.4±5.7 | 157.2±6.1 | 185.4±9.9 | 201.0±9.9 | 200.6±8.5 |
| ZnO-N | 155.2±5.7 | 159.6±4.7 | 178.2±8.2 | 190.2±3.5 | 198.8±3.7 |
| ZnO  Control | 153.2±8.5  144.0±4.8 | 164.2±7.4  149.6±4.0 | 185.0±7.4  172.6±2.5 | 190.8±7.3  182.0±4.5 | 202.8±5.4  199.6±5.3 |
